# Complete Structure of an Epithelial Keratin Dimer: Implications for Intermediate Filament Assembly

**DOI:** 10.1371/journal.pone.0132706

**Published:** 2015-07-16

**Authors:** David J. Bray, Tiffany R. Walsh, Massimo G. Noro, Rebecca Notman

**Affiliations:** 1 Department of Chemistry University of Warwick, Coventry, United Kingdom; 2 Centre for Scientific Computing, University of Warwick, Coventry, United Kingdom; 3 Institute for Frontier Materials, Deakin University, Geelong, VIC, Australia; 4 Unilever R&D Port Sunlight, Wirral, United Kingdom; Dalhousie University, CANADA

## Abstract

Keratins are cytoskeletal proteins that hierarchically arrange into filaments, starting with the dimer sub-unit. They are integral to the structural support of cells, in skin, hair and nails. In skin, keratin is thought to play a critical role in conferring the barrier properties and elasticity of skin. In general, the keratin dimer is broadly described by a tri-domain structure: a head, a central rod and a tail. As yet, no atomistic-scale picture of the entire dimer structure exists; this information is pivotal for establishing molecular-level connections between structure and function in intermediate filament proteins. The roles of the head and tail domains in facilitating keratin filament assembly and function remain as open questions. To address these, we report results of molecular dynamics simulations of the entire epithelial human K1/K10 keratin dimer. Our findings comprise: (1) the first three-dimensional structural models of the complete dimer unit, comprising of the head, rod and tail domains; (2) new insights into the chirality of the rod-domain twist gained from analysis of the full domain structure; (3) evidence for tri-subdomain partitioning in the head and tail domains; and, (4) identification of the residue characteristics that mediate non-covalent contact between the chains in the dimer. Our findings are immediately applicable to other epithelial keratins, such as K8/K18 and K5/K14, and to intermediate filament proteins in general.

## Introduction

Intermediate filaments (IF) are one of only three types of fibrous proteins in the human body. Keratins are the most numerous IF and are found in skin (e.g. K1/K10, K5/K14, K8/K18), hair (e.g. K86/K36, K82/K80, K71/K25) and nails [1,2]. In skin, keratin responds to and redistributes mechanical stresses across the cell, contributing to the skin’s elastic properties [3,4]. IF fibers comprise hierarchical assemblies, starting with the fundamental dimer sub-unit that self-organizes into higher-order sub-units (such as the tetramer and so forth) ultimately leading to the formation of the filament (Fig Aa in S2 File) [5–12]. Molecular scale insights into the interactions that drive and stabilize assembly of the higher-order sub-units are not clearly established but are much needed.

**Fig 1:**
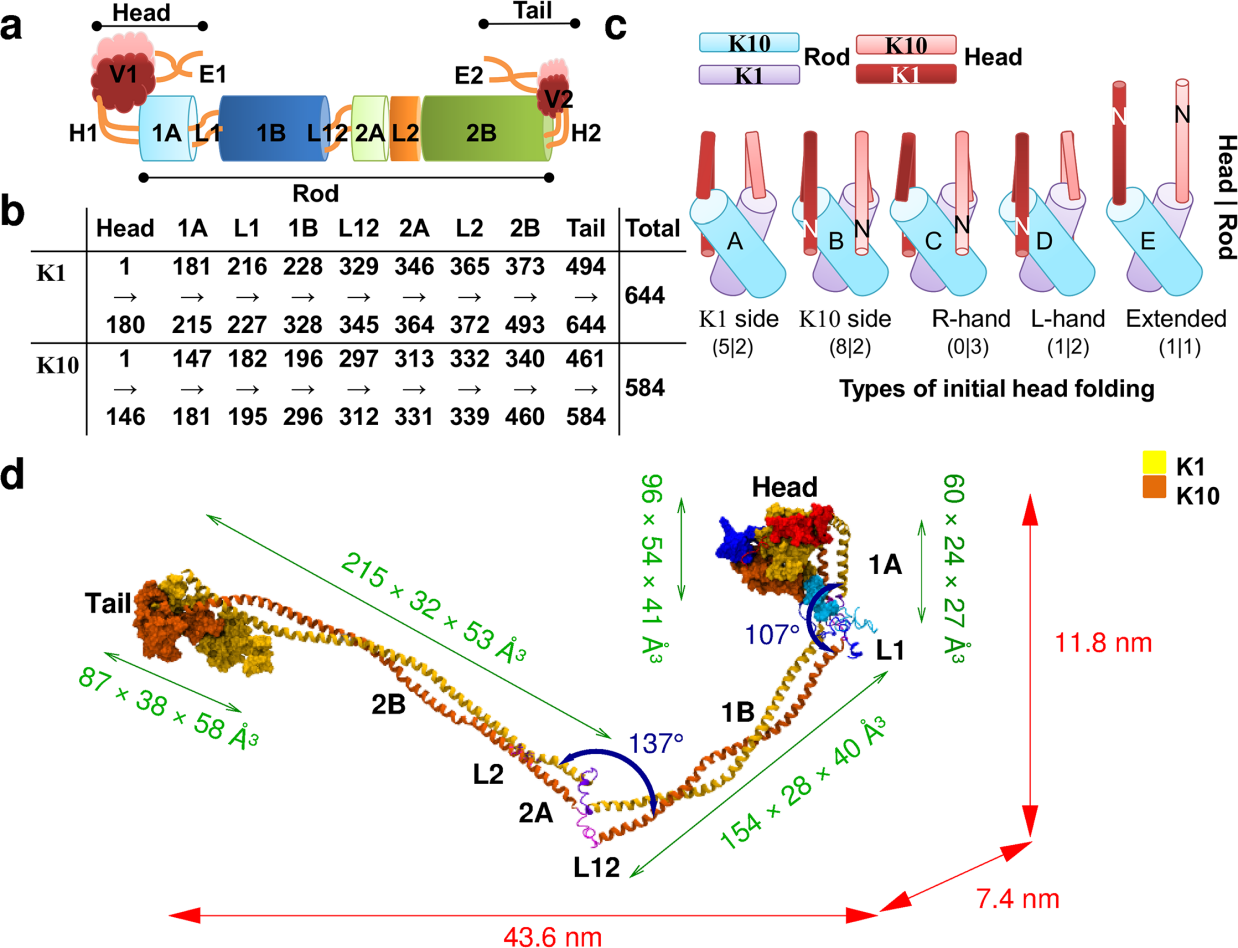
Structural models of the keratin dimer. (A) Schematic of the domain structure of the keratin dimer. (B) The number of residues in each subdomain. (C) Initial starting arrangements for the head domain, shown projected out of the 1A domain (values refer to the number of truncated/full dimer models considered). (D) An example of equilibrated dimer structure annotated with the mean principal component dimensions for each major domain and the hinge angle across the L1 and L12 linker.

Experimental evidence confirms that all IF protein dimers have the same tri-domain structure: a head, a central rod, and a tail domain [7,10] (Fig 1A). However, there remain a number of questions on the exact details of the dimer structure. These questions remain, despite advances in experimental characterization techniques, because of the substantive experimental challenges involved in gaining detailed atomistic-scale models at any level of the sub-unit hierarchy. Much of this experimental progress has focused on resolving the rod domain structure, which has extensive regions of coiled-coil structure. One key question regards how the conformation(s) of the largely uncharacterized head and tail domains impact on both the assembly and functional properties of keratin as a whole. For example, the second stage of hierarchical filament formation is thought to be the anti-parallel arrangement of dimers into a tetramer, which then propagate into protofilaments [7,9,13]. Intuitively, the interfacing between the highly structured rod domains should be the predominant driving force of sub-unit association. However, experimental data suggest that without the presence of the head domain, fibers do not form [14,15]. Experimental evidence further shows that the presence of the tail domain influences the width of the resultant fiber [16,17], and thus could play a role in fiber compaction. Additionally, experimental evidence indicates that head and tail domains play a critical role in water retention regarding the elasticity of the fiber [18]. Hence, in the absence of full, experimentally-derived detailed structural models of the rod, head and tail domains, molecular simulation can play a complementary role in connecting keratin structure with function.

Keratins form heterodimers; type I and type II keratin proteins intertwine in-parallel and in-register to give a central rod domain comprising, from the N- to the C-term, the 1A-L1-1B-L12-2A-L2-2B subdomains. 1A, 1B, 2A and 2B are coiled-coil subdomains, and L1, L12, L2 are the intervening linkers (Fig 1A). The sequence of the central rod domain is highly conserved amongst epidermal keratins and furthermore, shares a high similarity with other IF’s, such as the Type III homodimer vimentin. Some experimental data are available for keratin, however, there is relatively more experimental information available regarding the structure of the rod domain of vimentin. X-ray crystallographic techniques using a ‘divide and conquer’ approach have suggested that the vimentin rod is principally a left-handed coiled-coil [7,10,14]. However, the compartmentalized nature of this structure-determination approach does not rule out that some details, apparent only *via* studies of the *entire* dimer structure, may not have been captured. Furthermore it has highlighted that the 2A subdomain of vimentin may contain parallel strands rather than the left handed coiled-coil seen elsewhere [19,20]. The corresponding evidence for keratin is less complete, with crystal structure data available only for part of the 2B subdomain of K5/K14 [5]. In the absence of conclusive data, molecular simulations may offer a route to further refining the rod structure from the experimental data to reveal the chirality of the coiled-coil arrangement along the rod-domain, and improve the interpretation of the impact of this chirality on the higher-order sub-unit assembly and structure.

In contrast to the rod domain, the sequence of the head and tail domains varies in length (*e*.*g*. the head domains of K1 and K18 comprise 180 and 80 residues, respectively) and identity between different keratins. These domains are generally longer for the type II keratins (e.g. K1, K5, K8 *etc*.) than the type I keratins (K10. K14, K18 *etc*.). Epithelial keratin sequences typically possess high numbers of glycine residues and few cysteine residues, while trichocyte keratins contain relatively greater numbers of cysteine residues. Current understanding on the head and tail domains indicates that these correspond with intrinsically-disordered regions of the keratin protein [21]. Nonetheless, the overall size of the dimers, which in mature fibers is 44 nm [7], is thought to be approximately the size of the rod domain (40–50 nm) [9]. This may suggest that the head and tail domains are folded back onto the rod domain, as has been experimentally observed for the vimentin dimer head domain [22] while the corresponding tail domain was found to be flexible [23]. A sub-division of the head- and tail-domains of keratins has been proposed on the basis of sequence analysis alone [15,24–26], but this analysis has never been verified *via* experiment or simulation. This analysis suggested that the head domain could be divided into three subdomains, denoted as the end subdomain, E1, a variable glycine-rich subdomain, V1, and, a region of sequence homology close to the rod domain, H1 (see Fig Ab in S2 File). A second set of homologous residues was later identified in type II keratins [25], that correspond to the C-term residues of the proposed E1 subdomain. These conserved residues may thus be important in defining the boundaries of these proposed subdomains. On the same basis, the tail domain could be similarly sub-divided [15,24,26]. Presently, the tri-subdomain structure of the head and tail for keratin remains a hypothesis. The evidence base provided by molecular dynamics simulations of the keratin dimer may refine and characterize this tri-domain structure in the head and tail domains, giving vital clues into the dimer-level structural arrangement.

Previous work has reported the use of modeling to investigate the structure of IF proteins. Smith and Parry [27] visualized the three-dimensional arrangement of residues in the rod domain of the K1/K10, K5/K14 and K35/K85 keratin dimers by directly mapping the keratin residues onto the experimental structure of the vimentin rod. This analysis allowed a static assessment of the overall character of each face of the dimer rod, but could not accommodate for any structural changes due to the chemical differences in residues between keratin and vimentin. Neither could it provide any details on the head and tail domains. Molecular dynamics (MD) simulation offers an approach to model chemical interactions and can potentially yield physically-reasonable full structure predictions of IF proteins. Results of such molecular simulations have been reported previously for both the vimentin homodimer system [28–32], and recently, the trichocyte keratin heterodimer [33,34] (found in hair). These previous studies chiefly focused on the mechanical properties of the IF sub-units, rather than the structural details, and did not report local structural properties of the dimer at the residue level, nor did they provide any specific details on the structure and behavior of the head or tail domains. Furthermore, the majority of keratins are epidermal keratins; these have relatively little cysteine/methionine content compared with the trichocyte keratins, and hence the structure of the epidermal keratins relies less on disulfide cross-linking. This makes the structure prediction of epidermal keratins more challenging; not only are there fewer clues regarding points of contact in the IF sub-units (as would be indicated by the disulfide bridges in trichocyte keratins), but also, the sub-unit assemblies are structurally less rigid, owing to the lack of covalent linkages. Use of MD simulation to predict full structural details of epidermal keratin, including a characterization of the head and tail domain structures, as well as the rod domain chirality, has yet to be reported.

Here we detail the use of MD simulations to generate multiple, full, molecular structures of the entire K1/K10 heterodimer, including an analysis of the disordered head and tail domains, and demonstrate that these results are consistent with the existing data on IF structure. These keratins are found in the upper epidermal cells in the stratum corneum of human skin. From our statistical analysis of several structural models, we have elucidated common features of the keratin dimer, yielding the most probable conformational motifs. Although our simulations consider the keratin dimer in isolation, our findings aid conceptualization of the arrangement of essential structural regions of the dimer needed for forming the keratin filament. Key to this is the prediction of the local helical character along the *entire* rod domain using these models. Our results further enable an estimation of the spatial extents of the intrinsically disordered head and tail domains, which has not yet been achievable experimentally. Also, our results indicate that the characteristics of the head and tail domains concurs with their being a tri-subdomain structure, as has been hypothesized solely from sequence analysis. The 3-D shape and characteristics of these subdomains may aid elucidation of how the head domain confers interaction between dimers in the fiber assembly process. The tail domain, in comparison, was found by our models to be less well defined in shape and more conformationally labile, in agreement with the recent experimental measurements performed on vimentin.

## Methods

To generate complete structures for the K1/K10 dimer we performed a total of 25 all-atom MD simulations using a range of starting configurations. Because of the system complexity, we used a mixture of full dimer models (ten simulations), as well as truncated dimer models (fifteen simulations). The truncated system comprised only the first half of the dimer, spanning the head-1A-L1-1B domains. Atomistic-level experimental data, obtained for closely-related systems such as vimentin, provided a crucial basis for the construction our structural models (see below). While such data for the rod domain are relatively more mature than for the head and tail domains, very recent electron paramagnetic resonance (EPR) measurements for vimentin have provided vital clues regarding the arrangement of the head domain[22,23]. These data help to justify our choice for the initial arrangement of the head and tail domains in our simulations (see below for more details). For the highly structured core rod domain, this central domain of the dimer was initially configured in a manner as has been observed for a similar IF (vimentin), and so necessitated only moderate restructuring during simulation. Given that few details on the head and tail domain structure are known, we considered a range of initial configurations for the head and tail domains, to better sample the available conformations; we simulated these until stable structures were obtained. We obtained equilibrated structures of keratin by simulating the dimer in an implicit solvent environment (see Section C in S1 File for details). For further refinement, eight of the equilibrated dimer structures were then modeled in an explicit solvent.

### Constructing the initial models of the dimer

To model the K1/K10 dimer we constructed several initial starting structures and used these as a basis for further refinement during equilibration of the MD simulation. Each dimer was built by first constructing the head, rod and tail domains and then linking these three domains together to form the dimer (additional details are given in Section A and B in S1 File).

Since detailed structures of key regions of the rod domain (i.e. the coiled-coil domains) exist for a related IF, vimentin, we used these as a starting point for our model of the K1/K10 dimer. Comparison between vimentin and K1/K10 rod domain sequence shows that there is a 30% and 90% sequence similarity in amino acid identity and residue type, respectively, and is suggestive of similar structure. The crystal structures of the vimentin 1A (3G1E.pdb [19]), 1B (3UF1.pdb [20]) and 2A-L2-2B (3KLT.pdb [35] and 1GK4.pdb [36]) coiled-coil subdomains were obtained from the RCSB protein databank (PDB; http://www.rcsb.org/pdb/).

The initial configuration of the coiled-coil rod sub-domain of the K1/K10 dimer was generated by overlaying the K1/K10 sequence (obtained from the online IF database [37]) onto the experimentally-determined structure of vimentin (see Section B in S1 File for details) to build the corresponding K1/K10 1A, 1B and 2A-L2-2B coiled-coil subdomains (see Section B in S1 File for exact procedure). To create the full rod domain structures these coiled-coil subdomains then needed to be spatially translated, reoriented and then linked in sequential order using the sequences of the L1 and L12 linker regions as bridges. Each of the L1 and L12 linkers was constructed in the form of loops (see Section B in S1 File for exact procedure and Fig Ad in S2 File).

The head and tail domains of the K1/K10 dimer comprise 365 and 275 residues, respectively, making these the second-longest head and tail domains of any keratin. There are little direct data available on the structure of the head and tail domains of keratin or any other closely related IFs. However, recent site-directed spin labeling and EPR measurements for vimentin [22,23] suggest that the head groups are folded back onto the rod domain such that residue 17 of vimentin (in the head domain) is close to residue 137 (in the 1A subdomain). In some of our initial structures we assumed that keratin had similarly folded head and tail domains.

To generate these folded initial configurations of the head domain, the primary sequence of each chain was built as a β-strand, protruding from the N-term of the 1A coiled-coil subdomain in the direction of the long-axis of the rod domain (*y*-axis of the simulation cell). Each chain of the head domain was looped back onto the rod domain using a hairpin turn of nine residues (*ϕ* = −162.5°, *ψ* = 162.5° and *ω* = 180°, see Section D in S1 File for definitions of these dihedral angles). The folded initial configurations of the tail domain, which protrudes from the C-term of the 2B coiled-coil subdomain, were generated in a similar way as the head domain to fold back on to the rod domain. Finally, the head and tail domains (details described below) were added to the rod domain to produce the full initial structure of the K1/K10 dimer, like that seen in Fig Af in S2 File.

In our models we have made no allowance for the existence of disulfide bonds between cysteine residues, but instead allowed the sidechains to interact purely via non-bonded interactions. The K1/K10 dimer has a total of seven cysteine residues (specifics are given in Section H in S1 File) and are not highly conserved amongst keratins. In the rod and tail domains there are no suitable pairings for disulfide bonding, whereas the head domain contains a total of four cysteines. We did not explicitly include disulfide pairs in our models because there is currently a lack of atomistic-scale structural information to guide our selection of which pairs to link. Without such guidance, our selection of disulfide pairs might unduly bias the resulting structures generated from our simulations.

### Initial model configurations of the head and tail domains

The most challenging parts of the dimer to model, due to the unavailability of detailed experimental data, are the intrinsically disordered head and tail domains. To better sample the range of folded conformations of these domains we considered 25 different models, varying the initial configuration of these domains. In ten of our models we simulated the full dimer; for these we built the tail domain in the same configuration as the head domain. In our remaining fifteen simulations, we considered a truncated model, where we cleaved the dimer at the C-terminal end of the 1B coiled-coil subdomain and terminated each chain with a COOH group. To ensure that this artificial termination of the dimer did not cause the 1B coiled-coil subdomain to unravel during the simulation, a harmonic restraint was applied between the between the C_*α*_ atoms of the C-terminal residues in each truncated keratin chain (*i*.*e*. residues K1:328 and K10:296). A force constant of 1000 kcal (Å mol)^−1^ and an equilibrium distance of 14.37 Å (the distance measured in the initial model of the full dimer), were used for the restraint.

There are a multitude of possible ways that each head/tail chain can be folded back on to the rod domain. The construction of our initial models of each head and tail model was based on two key degrees of freedom: (1) the direction of the turn relative to the rod domain of the dimer, and (2) the position of the turn along the sequence of the head/tail domain of each chain. Five plausible folding directions were chosen for folding the head and tail chains back on to the rod domain which covered the entire range of possible orientations for the two chains. These were to fold: (A) both chains towards the K1 face of the rod domain; (B) both chains towards the K10 face of the rod domain; (C) each chain onto the opposite chain face of the rod (*e*.*g*. K1 onto K10 and *vice versa*); (D) each chain onto its own chain face of the rod; and, (E) fully extended away from the rod domain (*i*.*e*. not folded). A schematic of these different initial configurations is presented in Fig 1C for the example of the head domain.

Another possible variable in our set-up of the head and tail domains was the initial position of the hairpin turn as a function of location along the chain length. We explored up to six different positions of the hairpin turn for each of the orientations (A-E). Hypothesizing that the head domain of keratin may be folded as far back as vimentin, we started by placing the hairpin turn across the residues K1:103–111 and K10:86–94 (such that the equivalent keratin residues were next to each other as seen for vimentin from the EPR measurements [22,23]) for the head domain, and across the residues K1:534–542 and K10:498–506 of the tail domain. We then shifted the position of the hairpin turn on all the chains by an additional *n* residues (denoted as *n*-shift) towards the rod domain (where *n* = −5, 0, 3, 5 or 10). The combinations of different orientation and *n*-shift values produced the 25 different models. An overview of the 25 combinations used for the dimer models is given in Fig Ae in S2 File. To limit the complexity, in our full dimer models we have configured the hairpin of both the head and tail domains such that they were folded in the same direction. It was assumed that since the head and tail domains are located a sufficient distance apart, they cannot influence one another, and can be treated independently.

### Atomistic simulation of models using implicit solvent

We performed MD simulations using the NAMD simulation package [38]. The protein was modeled using the CHARMM27 all-atom protein forcefield with cmap terms included [39]. Since we considered the keratin in liquid water at ~ pH = 7, histidine was left unprotonated. The long aspect ratio of the protein and the extended initial configurations meant it was not practical to equilibrate the structure using an explicit description of the solvent. Instead, the Generalized Born Implicit Solvent model [40] was used. The solvent dielectric was set to 78 (that of water). No counter-ions were needed, as infinite boundary conditions were applied. The cutoff used for calculating the Born radii was 14 Å. The pair list distance was 17.5 Å and non-bonded interaction cutoff at 16 Å, with a switching function applied from 15 Å.

We took two approaches to initiating the implicit solvent simulations. In the first approach, the entire structure was energy minimized using the steepest descent algorithm. The velocities were then initialized to the thermostat temperature of 305 K before proceeding to the MD. In the second approach, the backbone atoms were fixed during the energy minimization. A second minimization was then performed with only the backbone C_*α*_ atoms restrained. Next, with these restraints still present, MD was performed for 30000 steps from initially at-rest molecules, using the same Langevin thermostat. Finally, from this point, full unconstrained MD was carried out.

Each dimer model was simulated for 100 ns and was determined as equilibrated, with a stable structure, when the root mean square displacement (RMSD) in residue positions had plateaued (see Section C in S1 File). From the equilibrated data, the final 20 ns of trajectory data for each model were used for the analysis.

### Simulation of models in explicit solvent

Following equilibration in implicit solvent, eight representative, equilibrated models (both the full and truncated dimer models) were solvated with 250,000–300,000 TIP3P [41] water molecules using the VMD [42] solvate tool. The system was charge-neutralized by randomly replacing 14 water molecules with sodium ions using the VMD autoionize tool. The dimer was placed in a cuboid periodic cell, with the long-axis of the dimer oriented with the cell *y*-axis, and the remaining volume filled with water, such that the cell dimensions extended 20 Å in the *y*-direction and 10 Å in the other directions on either side of the protein.

The explicit solvent simulations were initiated by energy minimizing the system with the protein backbone atoms held fixed in space. Next the backbone C_α_ atoms were restrained and MD performed for 3000 steps, from initially at-rest molecules, in the NVT ensemble using the same Langevin thermostat as for the implicit simulations. Next, a *NPT* simulation was performed for 5000 steps with restraints still placed on the C_α_ atoms. Finally, all restraints were removed for the remainder of the simulation. Here a Langevin piston was used, with a target pressure of 1 bar, a time period of 100 fs and decay of 50 fs. Group pressure was used. The inter-particle forces were calculated using a switching distance of 10 Å, a cut-off distance of 12 Å and pair list distance of 14 Å. Particle mesh Ewald with a grid spacing of 1 Å was used to treat the long-ranged electrostatic interactions calculation. Simulations were run to obtain 25 ns of equilibrated trajectory data.

### Analysis

In the study we use statistical analysis over all of our 25 structural models to gain insight into the most likely structural features for the keratin dimer. In this sense, we have classified the frames taken from our trajectories based on a range of geometric criteria as detailed below, and we then used these classifications to essentially coarse-grain the underlying potential energy landscape of these keratin dimer systems.

#### Properties of the coiled-coil subdomains

The structural properties of the coiled-coil subdomains were calculated using the same procedure as outlined in previous studies on the experimental crystal structure data of vimentin [19,35,36]. The central axis of the coiled-coil was determined using the method given in Strelkov and Burkhard [43]. In this procedure, the path *p*_*N*_(*i*) of the central axis of the α-helix of each peptide chain was calculated first (where *N* indicates which of the K1 or K10 chains is being considered, and *i* is the relative residue number to the N-terminus of the subdomain), with each *p*_*N*_(*i*) calculated from the C_α_ positions of four conservative residues with positions *i* − 1, *i*, *i* + 1 and *i* + 2 in the α-helical chain. The path of the central axis of the coiled-coil *P*(*i*) is then the mean of the paths of the two α-helices, *i*.*e*. *P*(*i*) = (*p*_K1_(*i*) + *p*_K10_(*i*))/2.

The local radius, *R(i)*, of the coiled-coil can then be calculated as half the separation distance between the centers of the two peptide chains at residue *i*, *i*.*e*. *R*(*i*) = (|*p*_K1_(*i*) − *P*(*i*)| + |*p*_K10_(*i*) − *P*(*i*)|)/2. Next, a dihedral angle, Ψ, made between the two vectors that connect the center of the α-helix of a given chain (*e*.*g* K1 or K10) to the central path of the coiled-coil, at *i* − 1 and *i*, around the path vector of the central axis between these (as given in Strelkov and Burkhard [44]). Calculation of the dihedral angle was repeated between *i* and *i* + 1 to produce four estimates (two each from K1 and K10). An average of these estimates then defines the local change in radial angle of each peptide chain at residue *i*. The local pitch, *λ*(*i*), was estimated from the change in distance along the central path, from *i* − 1 to *i* + 1, and the change in dihedral angle Ψ, using $\lambda (i)=\frac{1}{2}(|P(i+1)-P(i)|+|P(i)-P(i-1)|)\;(\Psi /2\pi )$.

The vector of the major axis of each coiled-coil was defined as the normalized displacement vector between the positions of the central axis at the 2nd residue and 3rd to last residue of the subdomain. The hinge angle, defined as that between coiled-coil subdomains, can then be calculated from $2\pi -{cos}^{-1}(\vec{u.}\vec{v})$, where $\vec{u}$ and $\vec{v}$ are the vectors of the major-axes of the two subdomains.

#### Globule axial displacement and radial orientation calculation

**Fig 2:**
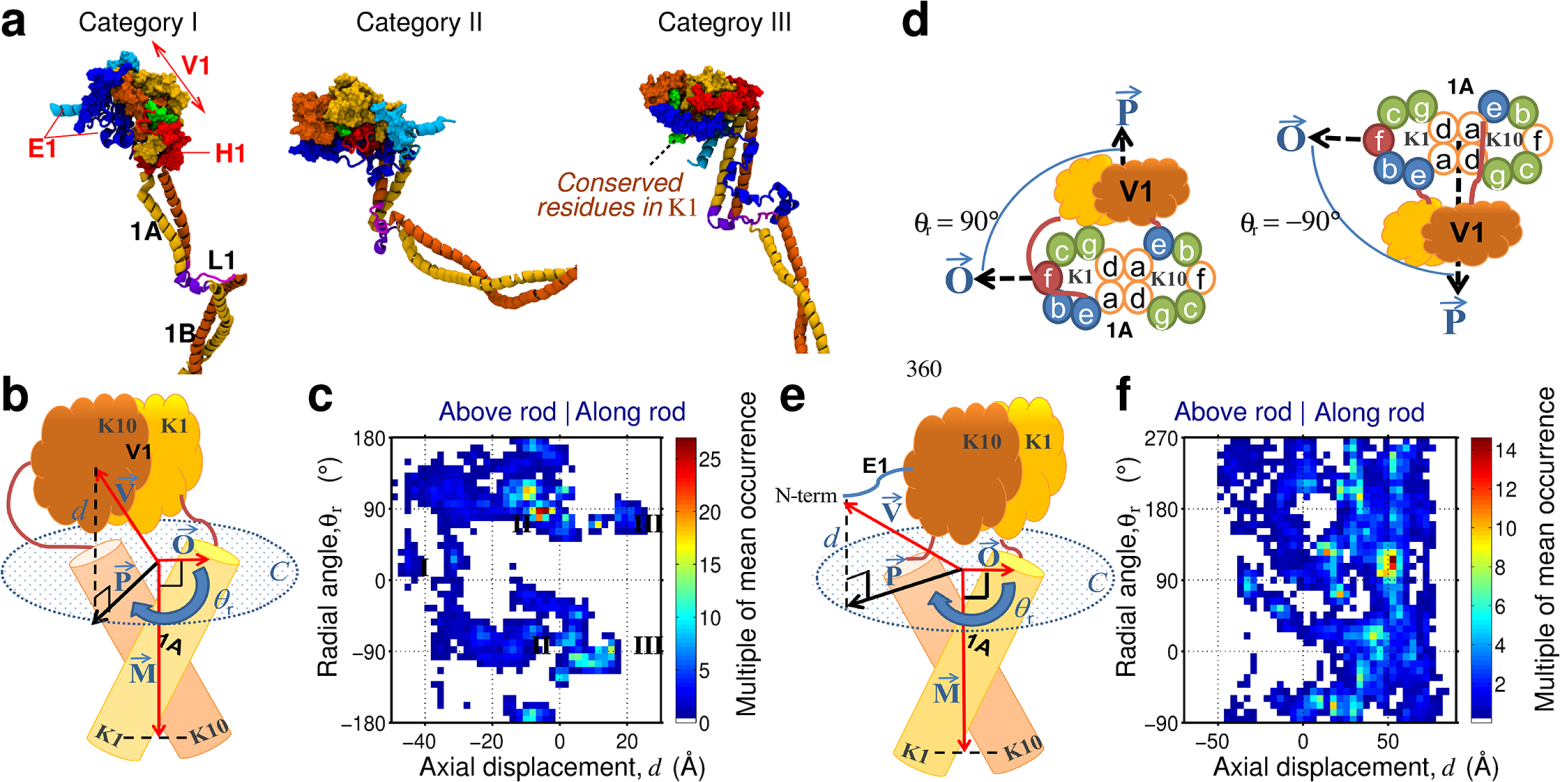
Positioning of the head domain. (A) Three exemplar positional preferences of the head domain. (B and E) Schematics of 1A and head domain showing key measurements made on V1 and E1 head domains (definitions of vectors are given in Methods). (C and F) plots of the occurrence of the positions taken by the V1 globule and N-term of the E1 head domain, respectively, with annotations referring to the examples. (D) top down diagrams illustrating the radial positioning of the head domain and the heptad positions of the 1A rod domain (the N-termini of the 1A starts at the *e*-heptad position and then runs clockwise into the page).

To calculate the axial displacement, *d*, and the radial orientation, *θ*_*r*_, of the V1 globule subdomain of a given chain (K1 or K10), we first defined its center-of-mass using the C_*α*_ atom positions of the chain found within the V1 globule. We then defined a vector, $\vec{V}$, between the V1 center-of-mass and the N-term of the 1A subdomain. The latter was defined as the 2nd residue position of the major axis of the 1A coiled-coil (as defined in the previous subsection). Second, we defined a normalized vector, $\vec{M}$, that pointed along the 1A coiled-coil major axis. We defined the axial displacement, *d*, as the projection of $\vec{V}$ onto $\vec{M}$, *i*.*e*. $\vec{V}\cdot \vec{M}$ (Fig 2B).

To calculate the radial orientation of the chain’s V1 subdomain, *θ*_*r*_, we constructed a plane C, defined as perpendicular to the 1A major-axis $\vec{M}$ and we considered the two N-termini (of K1 and K10) of the 1A subdomain to lie on this plane C. We also defined a normalized vector, $\vec{O}$, between the center-of-position of the K1/K10 N-termini of the 1A subdomains, and the N-terminus of the K1 1A domain (Fig 2B). From this we constructed a vector, $\vec{P}$, defined as the normalized projection of $\vec{V}$ onto plane C and thus calculated *θ*_*r*_ as the angle between the vectors $\vec{P}$ and $\vec{O}$, *i*.*e*. ${cos}^{-1}(\vec{P}.\vec{O})$ (Fig 2B).

**Fig 3:**
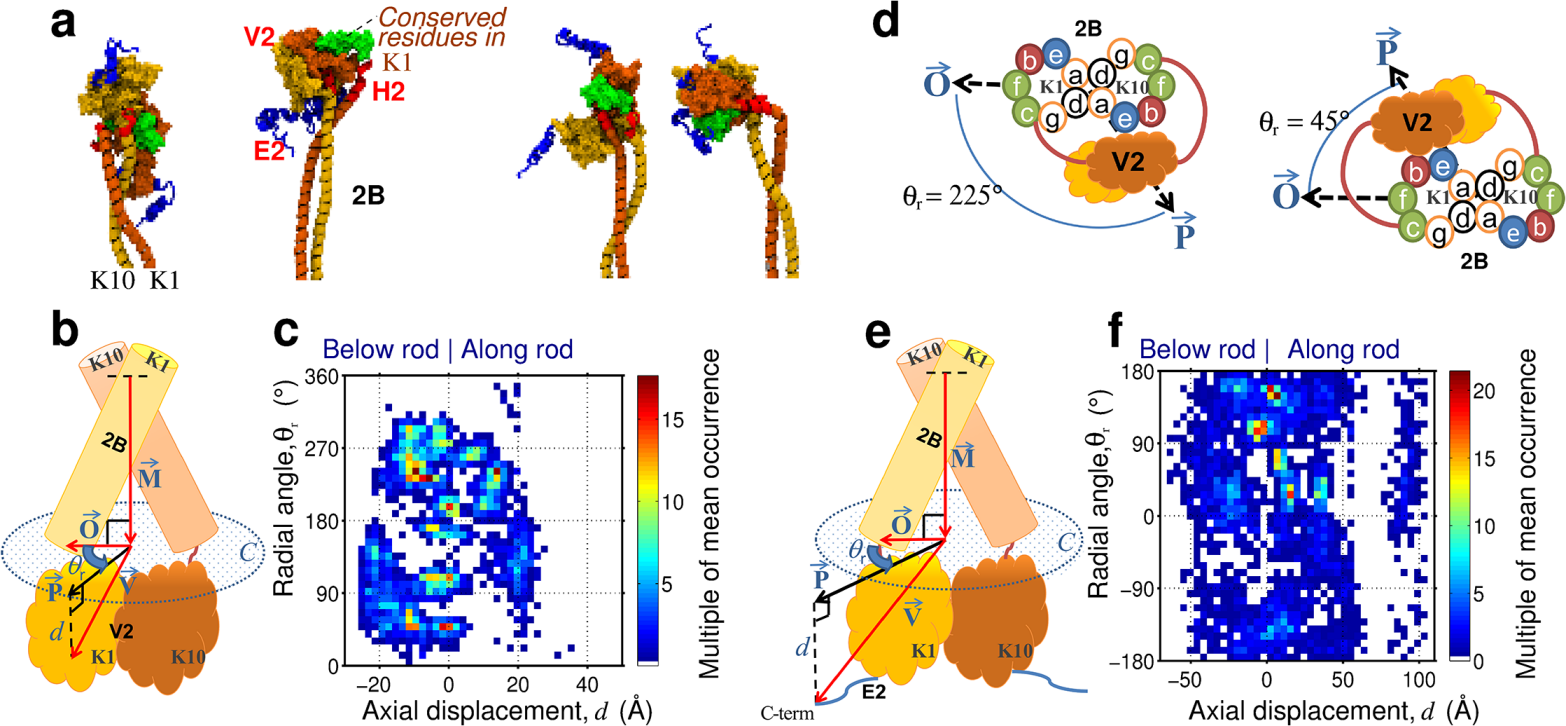
Positioning of the tail domain. (A) Exemplar positional preferences of the tail domain. (B and E) schematics of 2B and tail domain showing key measurements made on V2 and E2 head domains (definitions of vectors are given in Methods). (C and F) plots of the occurrence of the positions taken by the V2 globules and C-term of the E2 tail domain, respectively. (D) bottom down diagrams showing the radial positioning of the tail domain and the heptad positions of the 2B rod domain.

This procedure was repeated for the tail globule (substituting the residues of the V1 subdomain for those of V2 subdomain residues), and using the positions of the 3rd to last residue of the 2B subdomain as reference, see Fig 3B for how these definitions map onto the tail domain.

Finally, the position and orientation of the end termini of the head and tail domains were calculated using the above procedure and substituting the residues of the V1 subdomain for either the N-term or C-term residue of the head or tail domain, respectively (see Figs 2E and 3E for schematics).

#### Head and tail measurements

The radius of gyration was defined as the root mean square of the distances between individual backbone atoms of the head, tail domain or V1 subdomain and their center of mass. The end-to-end distance of the head and tail domains was defined as the distance between the C_α_ atom of the K1 (or K10) chain terminal residue (e.g. first or last residue, respectively) and the center of the N-term or C-term of the 1A and 2B subdomains, respectively.

#### Measurement of distance between pairs of residues

From each model the distance between the C_α_ atoms of a pair of residues was sampled uniformly in time and considered for analysis only if this distance was less than 12 Å. Residue pairs were classed into three types of interaction based on the chains involved: K1-K1; K10-K10; K1-K10. These were then used to calculate the frequency that a particular pair of residues are found to be close in these models. The results were normalized to account for the differences in residue numbers between truncated and full dimers.

We also measured the time average number and types of residue sidechain contacts present in each dimer model. Each specific side chain contact was classified depending on the pair of residue types present (as defined by the residue pairing matrix in Fig Fc in S2 File). To avoid double counting, only one type of interaction was measured for each sidechain pairing, which potentially involved two or more types of interactions. At any sample time contacts were said to be present if the distance between a pair of sidechain reference atoms was less than contact-type-dependent cutoff distance (these values are given in Fig Fc in S2 File) and added to a count. The probability of occurrence of a pairing was then determined by the total count divided by the total number of samples. For the pairings between the four polar residues, Ser, Thr, Gln and Asn, both potential side-chain sites for hydrogen bonding were checked. The average total number of contacts were calculated by summing up the probabilities over the K1-K1, K1-K10 and K10-K10 contacts. The values from these results were used to determine the coiled-coil side chain contact map.

## Results

In general, the equilibrated dimer structures had high aspect ratios, with mean principal components of 43.6 nm × 7.4 nm × 11.8 nm (Fig 1D), which are consistent with experimental values. Measurements of the average hinge angle made between the neighboring axes of the coiled-coil rod domains (see Fig Db in S2 File) indicated that the linkers L1 and L2 acted like flexible hinges, leading to deviation away from linearity for the dimer, with average angles between the 1A and 1B subdomains of 107°, and 1B and 2A subdomains of 137°. In all cases the head domain formed a single large globule, with a mean radius of gyration ~25 Å, (Fig Dd in S2 File and Methods). The average end-to-end distance of the chains of the head domain (see Methods for details) was found to be ~50 Å (Fig De in S2 File). The tail domain, in contrast, had a mean radius of gyration ~22 Å, and a mean end-to-end distance of ~45 Å (Fig Dd and De in S2 File). Herein, we detail results of our simulations with respect to the chirality of the rod-domain, the inter-chain interactions that drive association within the rod domains, and details on the domain structure(s) of the head and tail domains.

### Chirality of the rod domain

Since the rod domain is the most extensively experimentally studied part of IF dimer structure our analysis of the rod domain was conducted so as to be compatible with existing studies. First, we measured the local helical properties of the major coiled-coil subdomains of the rod domain, because these can be directly compared with the available experimental crystal structure data of K5/K14 [5] and vimentin [20,35,43]; these are the key metrics reported in these studies.

**Fig 4:**
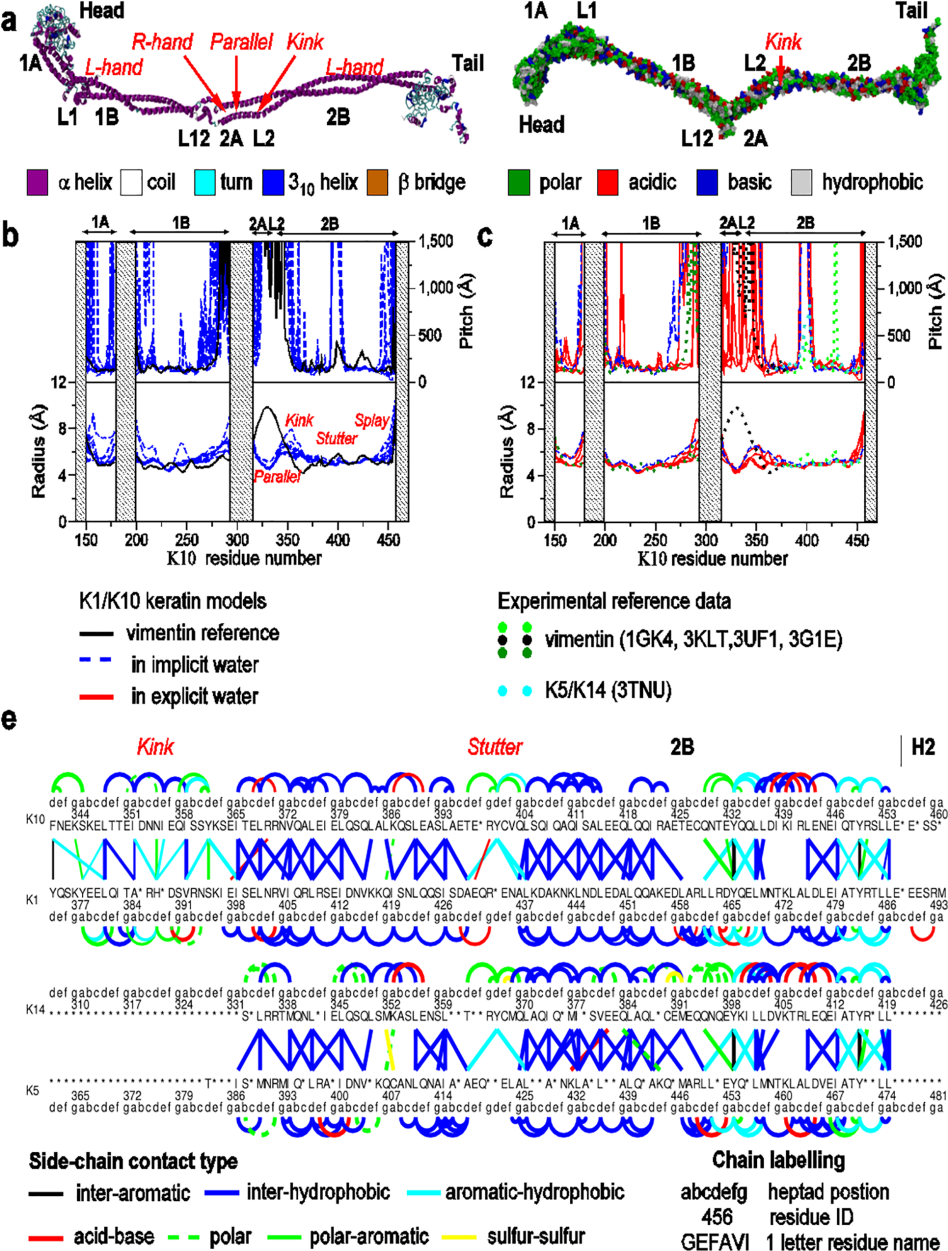
Rod domain structure. (A) Example final dimer structures with secondary structure shown on the left and residue type shown on the right. (B) Local radius and pitch of the coiled-coil subdomains 1A (left), 1B (center) and 2A-L2-2B (right) obtained from simulations using implicit solvent. (C) The same plot showing a comparison of four dimer models obtained using implicit solvent or explicit solvent and experimental crystal structure data of vimentin and keratin K5/K14. (D) comparison of the side chain contacts of the 2A-L2-2B rod domain, between our models of keratin K1/K10 in explicit water and the K5/K14 crystal structure. For clarity only contacts with at least a 0.5 probability of occurrence in the models are shown.

In our models the behavior of the coiled-coil local helical pitch and radius (Fig 4B and 4C) was overall convergent across our models. In Fig 4B we show the analysis from the dimer models in implicit water. For purposes of model evaluation, the solid line in Fig 4B indicates the local pitch and radius of the initial profile for the model prior to MD simulation. The difference between this and the final structures (dashed lines) demonstrates that our structural models were able to restructure to more appropriate forms for keratin.

In Fig 4C we provide a comparison of the further refined models of K1/K10 in explicit water (solid lines) and overlaid these with the experimental data for vimentin and keratin K5/K14 (dotted lines). Fig 4C shows that our model data agrees closely with both the vimentin data (as used to develop the initial model) and the more closely related keratin. This figure demonstrates the closeness between structures of the rod domain for the family of IF dimers with key properties in the coils-coil maintained, such as an average radius of 5 Å [20], a pitch of 170 Å [27] along with structural features in coiled-coil behavior, such as the three-residue stutter in the 2B subdomain (indicated by the increase in pitch of the models around residue K10:400). These features are highly conserved in IFs [7,43]. Also indicated on these figures is the retention of the 2B domain C-term splay (around K10:449), leading to an increase in both pitch and radius [35].

In Fig 4C we further compare the pitch and radius of the coiled-coils of four dimer models obtained using explicit solvation instead of implicit solvation. Re-solvating the models in explicit liquid water did not significantly alter the form of these coiled-coils: there were some differences in the more sensitive coiled-coil pitch (discussed below), but not the radius, compared with our implicit solvent data.

The pitch and radius of the 1A and 1B subdomains, and majority of the 2B subdomain, were consistent with a left-handed coiled-coil. The interface across the 2A, L2 and 2B subdomains has been challenging to experimentally characterize [20,35]; our structures showed that this region exhibited considerable variability (e.g. flexibility) in coiled-coil pitch and radius (around K10:340–360) both between models, and at different times for the same model. We identified the 2A and L2 subdomains as right-handed and parallel-chained (indicated by the high pitch values), respectively. This arrangement appeared to be enforced by the presence of the first two cross-chain pairings of internally-faced aromatic residues located after the L12 linker, at K10:325 and 339–340, that helped kept the chains parallel in this region. The parallel-chained coiled-coil continued from the L2 subdomain into the first few residues of the 2B subdomain. At K10:354–356 our models exhibited a range of radii for the coiled-coil, and an uncertain pitch value. Visual inspection of the structure confirmed that this region corresponded to a kink in the coiled-coil (as seen in Fig 4A), consistent with a similar feature observed experimentally in vimentin [20]. We identified the sequence stretches in the kink (*e*.*g*. K10:DNNI and K1:GRHG); the α-helical propensities for these motifs are unfavorable [20], and the heptad repeat of the coiled-coil places a basic residue in the *d*-heptad position (acid residues in similar positions were also shown to lead to such a kink [45]). These features may weaken the coiled-coil, causing the kink.

With good agreement between the model rod-domain profile and experimental data we next investigated the internal structure of the rod domains by quantifying the inter-chain side-chain contacts of the rod domain. Overall, coiled-coils were maintained by an inter-chain (K1-K10) interdigitation of hydrophobic residues. As a theoretical maximum, an ideal coiled-coil rod domain for keratin K1/K10 would involve 228 inter-chain side-chain interactions linking between the *a*- and *d*-heptad helical positions of the K1 and K10 chains. In practice, our simulations revealed on average 214 such contacts; of these, 80% (172 in total) were hydrophobic, with 12 of these due to aromatic ring contacts. Nine further contact pairs were found between acid and basic sidechains. This contrasts with the *intra*-chain sidechain interactions of the α-helical chains, where each have twice the number of electrostatic attractions (compared with the *inter*-chain case), given there are 19 out of the 197 observed K1-K1 interactions and 19 out of the 192 observed K10-K10 interactions (see Section F in S1 File for details). We profiled these sidechain interactions across the rod domains and compared these against the vimentin and K5/K14 crystal structures (using the same methods). Fig 4D shows a comparison between the 2A-L2-2B domain of the K1/K10 model and the K5/K14 experimental data. Here for the K1/K10 model we only show the side-chain contacts that occurred at least 50% of the time in our models. Despite some sequence differences between the two keratins, clear similarities in the internal structure can be seen.

### Head domain structure

**Fig 5:**
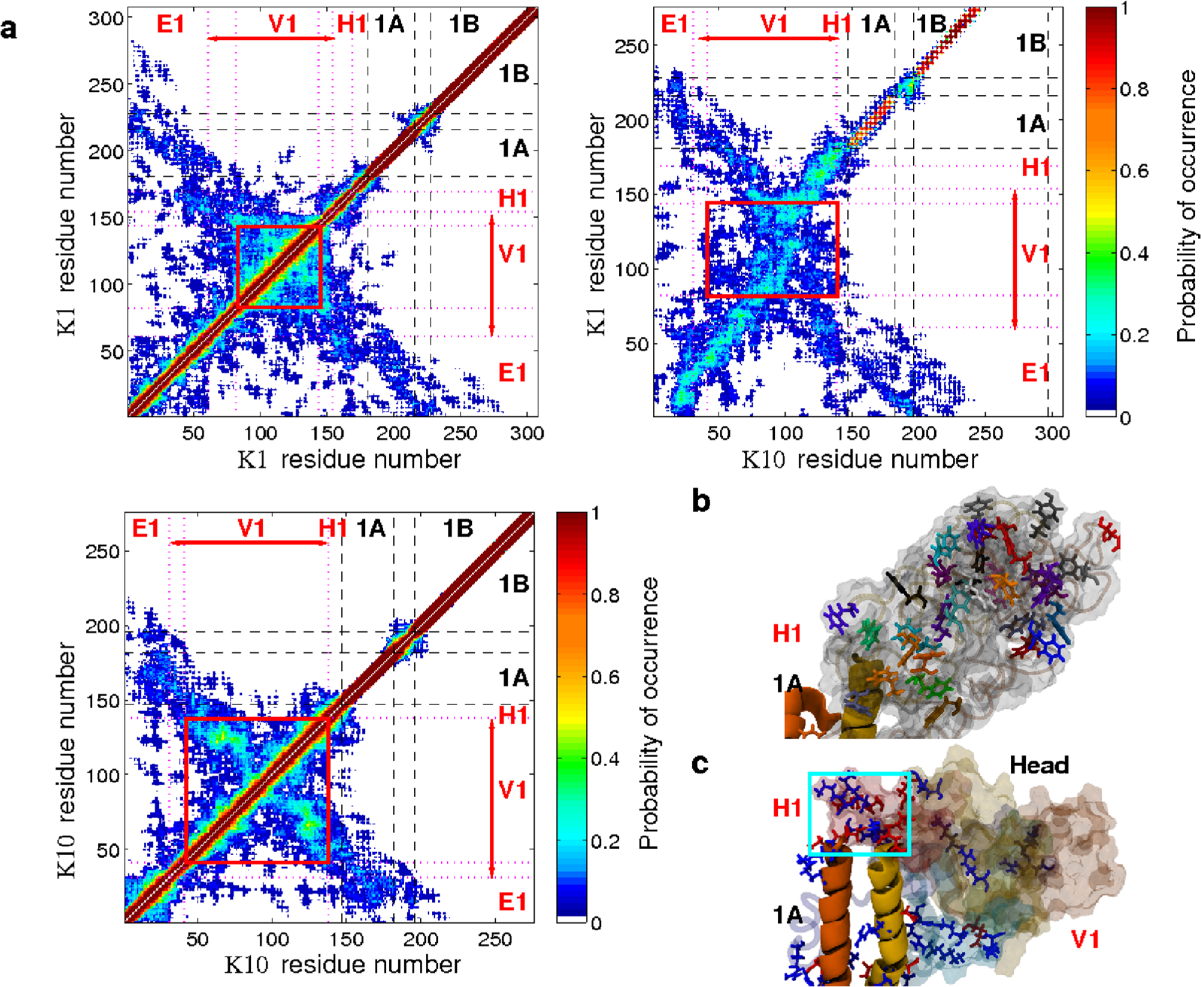
Head domain structure. (A) Combined contact plots for the head domain and part of the rod domain showing the probability of the **C**_**α**_ atoms of a pair of residues being separated by less than 12 Å. The dashed-lines show the boundaries between the head domain and rod subdomains, and dotted-lines indicate changes in the characteristics of the primary sequence (see Section E in S1 File). The letter annotations show which residues correspond to the rod subdomains 1A, L1 and 1B and our proposed head subdomains (H1, V1, E1). The red box indicates the residues that make up glycine loop motifs. (B) Snapshot of the head domain showing the aromatic and glycine loops contained within. (C) Snapshot of the head domain highlighting the acidic (red) and basic residues (blue) interacting between the rod and head domains. The rectangle indicates the location of acid and base pairing between the 1A rod domain and H1 head domain.

A key aim of our work was to obtain a comprehensive picture of the spatial extent and subdomain structure of the head and tail domains, generated via statistical analysis of their structure obtained from our 25 simulations. To indicate the detailed structure of the head domain, from each model we identified contacting pairs of residues (based on a distance cut-off between key atoms in the sidechains of the residue pair, see Methods) and produced composite plots of chain contacts, which colored each residue pairing by the overall probability of their occurrence in the equilibrated dimer models (Fig 5). For clarity, the residue pairs in our contact plots of Fig 5 were separated into three types of dimer interaction: two plots of intra-chain contacts e.g. K1-K1; K10-K10; and a plot of inter-chain contacts e.g. K1-K10. In these plots a region with high probability indicates a common feature in the dimer models.

We initially divided the contact plots by the experimentally known domain structure of the head and rod (e.g. Head, 1A, L1 and 1B subdomains), as marked by the black dashed lines and the black domain labels in Fig 5A. The statistical characteristics of the contacts within the head domain suggested a tri-subdomain structure (labelled on Fig 5 as E1, V1, and H1) that was consistent with the assignment implied from past sequence analyses of keratin [9]. To highlight these similarities we have taken the same sub-domain labeling of the head domain as has been previously proposed [9]. These subdomains were indicated by the less extensive contact seen between the V1 subdomain and the two other subdomains H1 and E1.

Our contact plots showed that residues K1:61–153 and K10:31–137 were typically in close contact and indicated these residues were part of a globular subdomain V1. This sub-domain encapsulates both the glycine loop motif (the spatial extents are indicated by the red box in Fig 5A), discussed below, plus further flanking residues that lay sequentially either side. In particular, integral to the V1 subdomain is a highly-conserved set of residues in K1, noted and defined in the sequence analyses as perhaps part of the H1 domain of K1, which were found to contribute a large number of the hydrophobic interactions associated with the head domain (totals shown in Fig F in S2 File). The high levels of K1-K10 interactions within this region indicates that the V1 globule could be considered as one unified structure, rather than two separate co-located chain folds formed from K1 and K10 separately.

The dominant feature of the contact plots is the V1 globule, featuring a 36% (107 residues) glycine content, which has been hypothesized to confer a ‘glycine loop’ structure [46]. Glycine loops are characterized by polyglycine repeats punctuated intermittently (every 3–6 residues in the head domain) by aromatic residues [46]. Glycine loop structures are thought to form via π-stacking of aromatic rings that are *neighboring* (but not sequentially adjacent) in the sequence [46]. Glycine loops have been attributed to a role in adhesion, arising from a ‘molecular velcro’ behavior [46], and could play a critical role in conferring the elastic properties of the keratin fiber. In contrast to the notion of ‘*neighbor-only*’ (in sequence) rings forming separate π-stacks, the regions highlighted by rectangles in Fig 5A indicate a more complex hierarchical 3-D arrangement of loop structures.

We identified the types of sidechain interactions in the head domain (see Section F in S1 File and Fig F in S2 File); on average each ring interacted with two of its aromatic neighbors, suggesting the aromatic residues are interlocked in loop-like structures. Furthermore, most hydrogen bonds in the head domain were found between backbone atoms, suggesting that the poly-glycine stretches were folded in close proximity (the distribution of the number of hydrogen bonds within the dimer are given in Fig G in S2 File and Section G in S1 File). A representative arrangement of glycine and aromatic residues is shown in Fig 5B.

### Relative Position and Orientation of the Head and Tail Domains

Having identified a sub-domain structure to the head domain where the K1/K10 chains are largely globular rather than extended, we next determined whether the head domain pointed towards the rod domain (and hence interacted with the rod) or extended away from the rod domain (and hence would be expected to be more mobile). We investigated the positional and orientational preferences of the head domain relative to the rod, and identified from the models three categories of head domain spatial position, depending on the statistical location of the head domain relative to the 1A subdomain of the rod (examples of each category are shown in Fig 2A): Category I–with the head domain projected out along the long axis of the dimer, beyond the N-terminal end of the 1A subdomain; Category II–with the head domain at right angles to the top corner of 1A; or Category III–with the head domain folded down onto 1A. To determine if the V1 globule showed any positional preference, we measured the axial displacement, *d*, and the radial orientation, *θ*_*r*_, of the chain’s center of mass of the V1 subdomain (see Methods). A positive value of *d* indicates the globule is positioned above the N-term of the 1A domain (Category I); similarly, a negative value of *d* indicates the globule is located at a point below the N-term of the 1A domain (Category III, Fig 2B). In terms of radial orientation, *θ*_*r*_ = 0° indicates the globule is positioned on the K1 side of the dimer, while *θ*_*r*_ = 180° indicates the globule is positioned on the K10 side of the dimer. Similarly, values of *θ*_*r*_ = ±90°, indicate the globule is positioned between the K1 and K10 chains (Fig 2D). In Fig 2C we plot the occurrence of *θ*_*r*_ *vs*. *d* for the V1 subdomain by superimposing the data from K1 and K10.

The most likely values of *d* and *θ*_*r*_ (Fig 2) show that the K1/K10 models favors the head domain to be positioned roughly at right angles with the rod domain (Category II), with the V1 subdomain resting between K1 and K10, and anchored to the rod via the H1 subdomain (also see Fig D in S2 File). This anchoring of the head domain followed the establishment of electrostatic sidechain interactions between N-term of 1A domain and charged residues in the H1 subdomains, shown in Fig 5C. In contrast, for instances when the axial displacement of V1 globule corresponded to Category I, no orientational preference was featured (a wide range of *θ*_*r*_ values were supported).

To identify whether the termini of the head domain (e.g residues 1 of K1 and K10) were anchored onto the rod or were free to move, we calculated preferences for *d* and *θ*_*r*_, for the N-term residue of the E1 subdomains in K1 and K10 (Fig 2F). Unlike V1, these residues showed no radial preference but did show a preference for the N-term of the head domain to be folded back towards the rod domain, suggesting a loose association. The contact between E1 and the rod (details given in Fig F in S2 File) showed a variety of contact types, with a large contribution from electrostatic interactions. This variety, and thus redundancy, of residue-contact types may be essential to maximize opportunities for establishing non-covalent contact between E1 and the rod.

**Fig 6:**
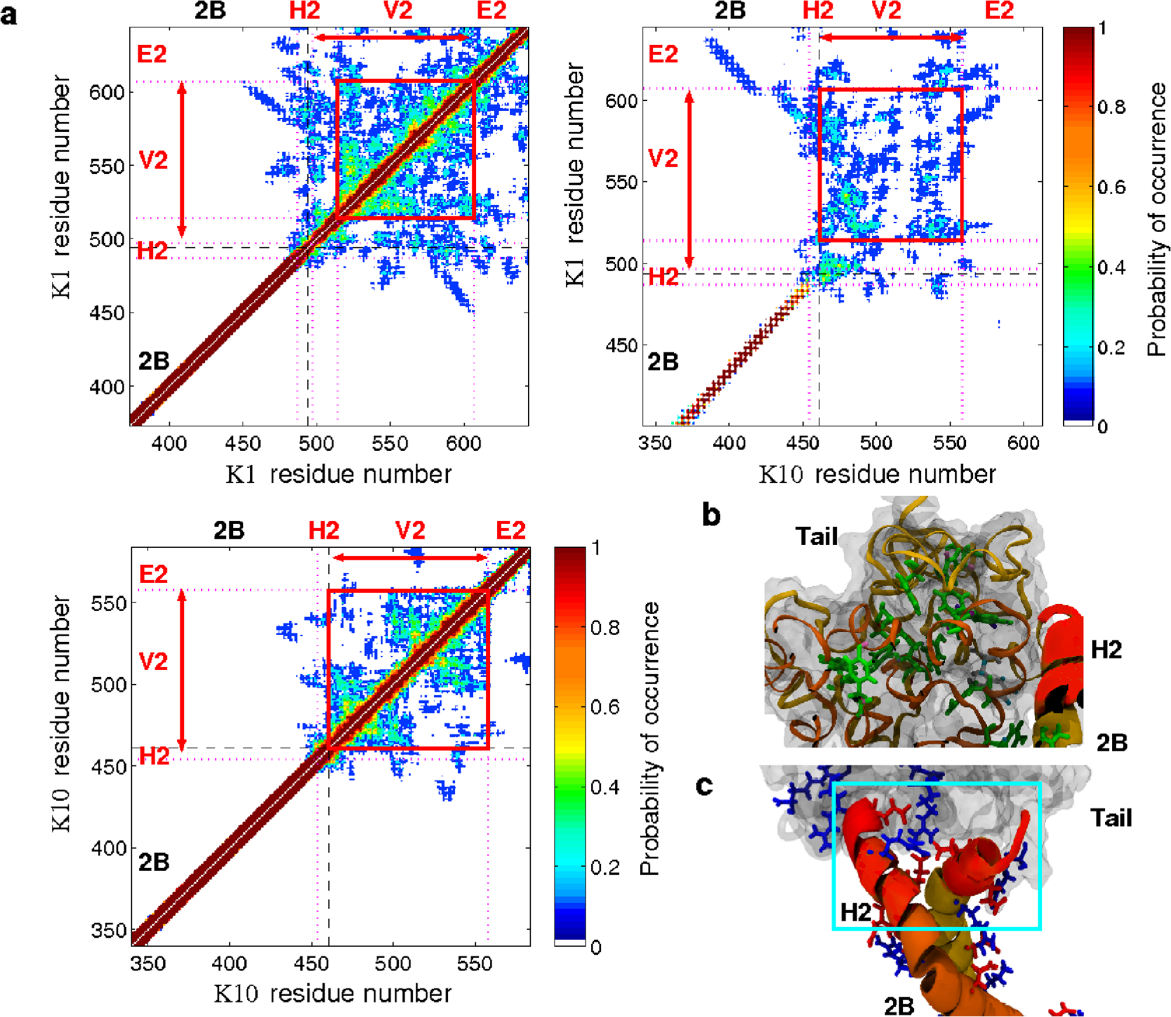
Tail domain structure. (A) Combined contact plots for part of the rod domain and tail domain showing for the probability of the **C**_**α**_ atoms of a pair of residues being separated by less than 12 Å. The dashed-lines show the standard boundaries between the rod domain and tail subdomains, and dotted-lines indicate changes in the characteristics of the primary sequence (see Section E in S1 File). The letter annotations show which residues correspond to the rod subdomains 2B and our proposed head subdomains (H2, V2, E2). (B) Snapshot of the tail domain showing the aromatic (in green) and glycine loops (yellow ribbon) contained within. (C) Snapshot highlighting the acid (red) and basic residues (blue) interacting between the rod and tail domains (inside box).

The tail domain presented the same types of questions on its structure as the head domain and thus we conducted similar statistical analyses as was done (see above) for the head domain, which is shown in Fig 6. Superficially, the visual appearance of the tail domain of K1/K10 appeared analogous to the tri-subdomain head domain. Fig 6A shows the residue pair contact plots corresponding to the rod and tail domains 2B-H2-V2-E2. Black dashed-lines demarcate the 2B rod domain and tail domain structure. We divided the tail into three subdomains (H2, V2, E2) based on characteristics of the primary sequence (Figs A and E in S2 File). Again, subdomain labeling was chosen to match previous sequence analyses studies of keratin. The contact plots of Fig 6A and the measured absence of K1-K10 intra-chain contacts suggest that the final seven residues of each chain of the 2B rod subdomain rather belong in the tail domain. This is because there is less structural coherence here than for the rest of the rod, further indicated by the lower probabilities in contact pairing found in H2 than the coiled-coil 2B rod domain (see Fig 6A). At this point in the sequence there are multiple glycines, potentially increasing the backbone flexibility relative to elsewhere in the rod. Previously, this sequence stretch had been described as a 7–10 residue splay in the C-term of the 2B rod domain [7]. Instead, our data suggests these residues are described as part of an H2 subdomain (residues K1:487–497 and K10:453–460).

The central V2 globule subdomain of the tail (K1:497–607 and K10:461–558), consists chiefly of glycine loop motifs, with 6–12 mainly glycines between pairs of aromatic residues. Analysis of the sidechain interactions within the tail showed that each aromatic residue is on average paired with one other (Fig F in S2 File), supporting fewer and much larger glycine loops in the tail, in contrast with the more complex arrangements in the V1. The hydrophobic patch of residues, K1:497–513, (previously described as the H2 domain in K1) appeared in the contact plot to be better described as part of the V2 subdomain. There are fewer interactions in the tail domain than in the head domain (see Sections F and G in S1 File). This may explain why the size and shape of the tail domain exhibited far more structural variability compared with the head domain. Experimentally this has been shown to be the case for vimentin [23].

In Fig 3B and 3C we show the positioning and orientational preferences for each chain of the V2 globule subdomain, as a function of the radial angle, *θ*_*r*_, and the axial displacement, *d*, of the V2 center-of-mass relative to the second to last residue of the 2B coiled-coil (see Methods). The position of the tail domain is less well defined than that of the head domain. This, along with representative snapshots of the tail structures (Fig 3A), suggests that the two chains form distinctly separate globular structures that are in contact with one another, in contrast to the single, unified globule as seen for the head domain. The spatial positioning of the V2 subdomain showed more variability (i.e. lower probabilities) compared with that for V1, and with a preference for both globules favoring either the K1 side or the K10 side (Fig 3D **)** centered about the end of the rod domain. Similar analysis for the C-termini of the E2 subdomain (Fig 3E and 3F) showed that the C-termini had a preference to be positioned at or below the C-term of the 2B subdomain and on the K10 side (*θ*_*r*_ = 120°), i.e. at the tip of the rod rather than folded back. The greater variability in this distribution, relative to E1, further supports the notion that the E2-termini are held more loosely to the rod compared with E1.

## Discussion

**Fig 7:**
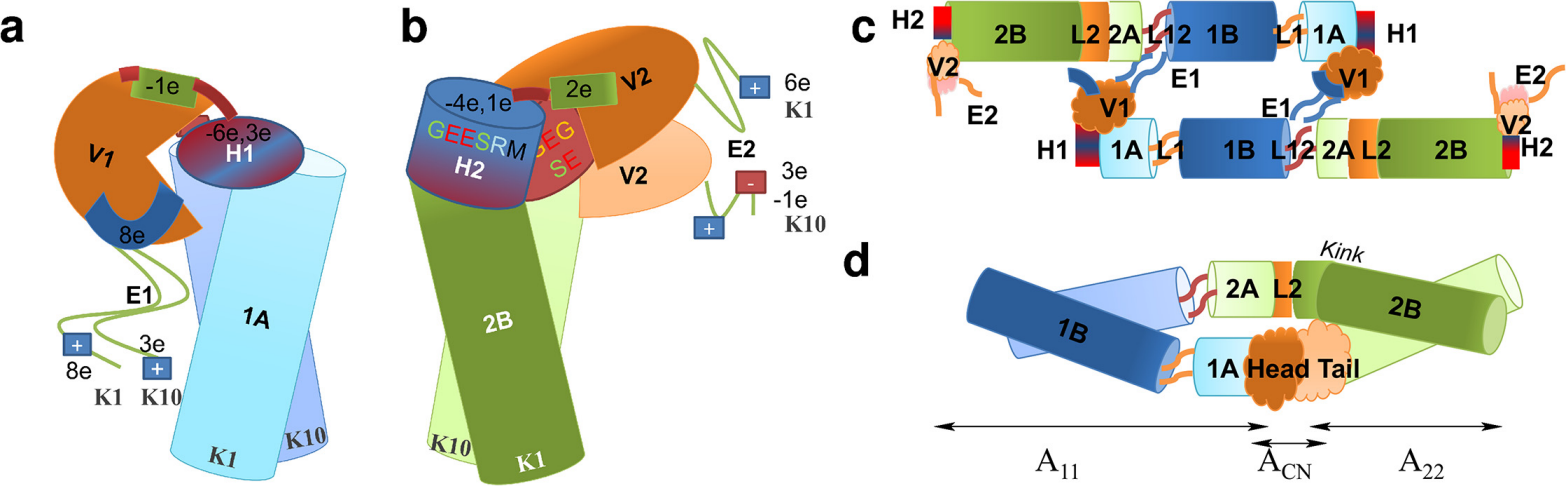
Proposed structural models. (A and B) Models of the head (A) and tail (B) domains with annotations indicating the local electrostatic polarity. (C) Proposed form for the A_11_ tetramer. (D) Proposed stutter-twisted form for the protofilament.

Our results suggest that the K1/K10 dimer features a kinked central rod domain with a complex helical twist and is capped by a head domain that is folded back down the rod and a tail domain folded more loosely to the side. Based on our findings, we propose model structures for the head and tail domains of K1/K10. The head domain, Fig 7A, comprises a conserved core surrounded by polar and aromatic residues, with a base-rich face projecting out of the lower surface towards the rod domain, and a charged face capping the rod. The tail domain, Fig 7B, features less electrostatic attraction, fewer glycine loops, and is much less structured. At the base of these globules is a set of conserved residues that lead to the charged end of the rod. These contrasts in head and tail structure emphasizes their different roles in filament assembly, where the head domain may act as an anchor, through which two keratin dimers might co-locate in registry, while the tail may help align the rod domains of the two dimers.

Aggregation of aromatic residues as seen in our models, via π-stacking, may be important for maintaining and restoring the globular nature of these head and tail domains, when not under mechanical strain. Such behavior is also seen in available crystal structures of the tail domain fragments of the IF proteins lamin (1IFR.pdb) and in blood fibrin (3H32.pdb). Unique to the K1/K10 dimer is the large number of glycine residues present in the head and tail domains. These may be required to give keratin sufficient elastic properties that allow these domains to expand when under tension. Such sequences are also present to a lesser extent in the K5/K14 and K8/K18 keratins, which are also found in the skin, but not in trichocyte keratins (as found in hair) or other IF proteins which may not have such such high elasticity requirements. Another feature that is not conserved amongst the IF proteins, but is present in K1/K10, is the overall basic character of the head and tail domains. In other IF proteins the head and tail domains are highly charged, but their characters range between basic (including many of the keratin head domain), acidic and mixed charge, suggesting that these are specific for the IF.

Our models of the rod domain indicate several kinks and stutters in the coiled-coil structure. These occur when the internal structure is disrupted from the interdigitation of an ideal coiled-coil. For example, in the 1B domain there are several places where a pair of opposing IF conserved, charged sidechains occupy the a- or d-heptad position of each chain (located at K10: 266, 245, 290 and the equivalent in K1). These features may be necessary for providing areas of interfacial registry between two dimers, through interlocking of the dimer rod profiles, which then induces the correct staggering of dimers within the tetramer structure.

In a keratin fiber, several dimers must hierarchically associate such that the central rods of neighboring dimers align. Experimental evidence suggests four arrangements of dimer pairs [7], denoted A_11_, A_22_, A_12_ and A_CN_. Filaments are thought to exhibit a right-handed supertwist in the protofilaments, which are formed from tetramers [7,13] and thus suggest a rope-like hierarchical structure, like that proposed by Utiu *et al*. [47] Therefore, the dimer pairs could be right-hand twisted to maximize interfacial contact area, analogous to the coiled-coils in the dimer [44]. If such a twisted structure was present, then participation by the parallel chains in the 2A subdomain would seem unlikely. Moreover, the bulky head and tail domains found adjacent to the 2A subdomain in the A_CN_ tetramer and hexamer (combining A_11_, A_22_ and A_CN_) structure would also seem unsuitable candidates for such twisting. Thus, for such a model to be plausible, we suggest that this region must feature a break from the twisting between dimers, allowing the head and tail to interface (Fig 7D), as seen in our models around the 2A-L2 domain. Such a stutter in the twisting may also necessitate a kink at the beginning of the 2B subdomain (as seen in our models), as the profile of the dimer would need to abruptly change from being non-helical in the 2A-L2 subdomains back to helical in the 2B subdomain (if the A_22_ interface is also in a twisted profile). A similar kink is not required between either the 1B and 1A, or 1B and 2A subdomains (Fig 7D) as the more flexible L1 and L12 linkers can perform this role. Experiment shows that when flexibility is removed from the L1 linker, by modifying to an α-helix, then fiber hierarchy cannot extend beyond the unit-length filament and A_CN_ end-to-end dimer association is disrupted [14].

In the keratin filament, it was previously observed that the head lies adjacent to the L12 linker [7,15], an acidic region of the sequence. At neutral pH, the head carries an overall charge +15e, mostly located in highly mobile E1 subdomain, but also in part of the V1 subdomain (see Fig 7A). In the fiber, our dimer model would allow these regions of the head domain to support substantial electrostatic interactions with L12 of a neighboring dimer (see also Kreplak *et al* [9] regarding vimentin) while allowing the bulk globule of the head domain to align with the 2A domain. In our model the end-to-end distance of the head is largely determined by the few N-term residues in E1 that are not in the V1 globule; the head domain is found to extend up to 50 Å back along the rod domain, sufficient to align the lower segment of the 1A subdomain with L12. Thus, by forming a non-covalent anchor between the head domain of one dimer and the L12 linker of the second dimer, the 1B rod domains of both dimers are able to align in an anti-parallel fashion, as in the A_11_ interface (see Fig 7C). Full alignment of the rods may then be satisfied *via* mutual twisting.

In summary, our MD simulations provide the basis for a more detailed and complete atomistic structure of the K1/K10 dimer, and suggest details for the unstructured head and tail domains that have thus far remained elusive to direct experimental characterization. Our models provide a useful reference structure of the dimer, in particular a complete profile of the core rod domain, for future experimental studies, while agreeing well with existing studies in the literature. It is anticipated that the structural models provided in our study will provide a valuable foundation for future experimental investigations of keratin structure at the atomistic level. In particular, application of x-ray crystallographic techniques (along the lines of the ‘divide and conquer’ strategy used previously for vimentin) would be extremely valuable for comparison with our predicted structures for the K1/K10 rod domain, while EPR measurements, alongside our predictions, would be enormously helpful in resolving likely structures of the head and tail domains. The good level of overall residue conservation found within all IFs means that our approach and findings will translate to give detailed insights into the dimer structures of IFs in general, particularly of the rod domain. Additionally for keratins, as several sections of the sequence of the head domain are conserved within keratins, our models suggest that these regions are key to defining the emergent sub-domain structure.

## Supporting Information

S1 File Supporting Additional Methodology. **Section A**, Models Used. **Section B**, Modeling of the rod domain. **Section C**, Equilibration of the dimers. **Section D**, Ensemble properties of the dimer models. **Section E**, Dividing the head and tail domains into a tri-domain structure using keratin informatics data. **Section F**, Sidechain Contacts. **Section G**, Hydrogen and Hydrogen-π Interactions. **Section H**, Sulfide Residues.
(DOCX)

S2 File Supporting Figures. **Fig A**, Keratin model building. **Fig B**, Evolution in structure of the truncated dimer models. **Fig C**, Evolution in structure of the full dimer models. **Fig D**, Statistical geometry of the dimer. **Fig E**, Definition of the subdomains of the head and tail. **Fig F**, Contacts between amino acid side chains. **Fig G**, Details on Hydrogen-bond interactions.
(DOCX)

S3 File Supporting data. Atomic coordinates of the final structures of our truncated dimer models.
(TXT)

S4 File Supporting data. Atomic coordinates of the final structures of our full dimer models.
(TXT)
