# Supplementary material for: Complete Structure of an Epithelial Keratin Dimer: Implications for Intermediate Filament Assembly
Source: PLoS One. 2015 Jul 16;10(7):e0132706. doi: 10.1371/journal.pone.0132706 (PMC4504709; doi:10.1371/journal.pone.0132706)
Supplement: S1 File — Section A, Models Used. Section B, Modeling of the rod domain. Section C, Equilibration of the dimers. Section D, Ensemble properties of the dimer models. Section E, Dividing the head and tail domains into a tri-domain structure using keratin informatics data. Section F, Sidechain Contacts. Section G, Hydrogen and Hydrogen-π Interactions. Section H, Sulfide Residues. (DOCX) [file pone.0132706.s001.docx]

S1 File: Supplementary Methodology for the Manuscript Entitled:

Complete Structure of an Epithelial Keratin Dimer: Implications for Intermediate Filament Assembly

David J. Bray^1,2^, Tiffany R. Walsh^3*^, Massimo G. Noro^4^, Rebecca Notman^1,2*^

^1^Department of Chemistry University of Warwick, Coventry, UK

^2^Centre for Scientific Computing, University of Warwick, Coventry, UK

^3^Institute for Frontier Materials, Deakin University, Geelong, VIC, Australia

^4^Unilever R&D Port Sunlight, Wirral, UK

*Corresponding author

Email: [r.notman@warwick.ac.uk](mailto:r.notman@warwick.ac.uk) (RN);tiffany.walsh@deakin.edu.au (TRW)

# Models used

Our study consisted of simulations of 25 model dimers, starting from a range of initial head and tail configurations. Fifteen of the models were of the truncated dimer and consisted of just the head and upper rod (1A and 2B subdomains) domains of keratin. **Figure Ae in S2 File** gives the initial arrangements for the head domain used in the truncated models. The remaining ten models were of the full dimer, where the head and tail domains were configured as given in **Figure Ae in S2 File**. Each dimer configuration given in the Tables was denoted by a letter, that corresponds to the A-E categories for the head and tail domains given in the main article, and a parameter *n*, which gives the relative residue position of the nine-residue turn in each chain of the head and tail domain, as described in main article. An example snapshot of the initial set-up of the dimer with the head and tail folded back onto the rod domain is shown in **Figure** **Af in S2 File**.

# Modeling of the rod domain

The initial structural form of the main coiled-coil subdomains of the rod domain were obtained by matching the equivalent part of the backbone of each peptide chain (K1 and K10) to the crystal structure data of vimentin, as detailed in the main text. Data on the 2A-L2-2B coiled-coil subdomains had been resolved in two separate studies (that overlap across seven residues [1,2]). The residues of the 2A, L2 and the 1st to 42nd or 44th residues of the 2B domain were obtained from 3KLT.pdb (vimentin residues 264-332/334 [1] and the remaining 43nd/45th to 121st residues of the 2B domain were obtained from 1GK4.pdb (vimentin residues 333/335-411[2]). Half the full dimer models were constructed using the 42nd residue of 2B subdomain as the marker, and the remainder used the 44th residue; none of the structural features were influenced by this choice.

Each crystal structure fragment comprised data on two identical chains, A and B, where both chains A and B are required to form a single vimentin homodimer. As keratin is a heterodimer, *i.e.* each chain has a different primary sequence, we chose to overlay the sequence of K1 onto chain A and the sequence of K10 onto chain B.

This was done by obtaining from the vimentin crystal structures, the bond angles and dihedral angles of the protein backbone for each residue in each chain. These bond and dihedral angles were then used to build the corresponding K1/K10 1A, 1B and 2A-L2-2B coiled-coil subdomains with the vimentin residues substituted for K1/K10 residues using the following procedure.

We began by generating the positions of the backbone atoms of each subdomain. Standard bond lengths of $|N-C_{\alpha}| = 1.457$ Å, ${|C}_{\alpha}-C| = 1.529$ Å, $|C-N |= 1.330$Å and $|C=O| = 1.240$ Å were assumed throughout. The scheme for generating the positions is illustrated in **Figure Ac in S2 File**. Starting with a fictitious C atom and the N-terminal N atom (labeled 1 in **Figure Ac in S2 File**), the position of the next backbone $C_{\alpha}$ atom (2) was then generated to satisfy the bond angle $\theta_{\mathrm{CN}C_{\alpha}}$. The subsequent $C$ atom (3) was then positioned to satisfy both the bond angle $\theta_{NC_{\alpha}C}$ and the dihedral angle $\phi$. The positions of subsequent backbone atoms were then generated by placing N, C_α_ and C atoms sequentially one after the other, using the positions of the preceding three atoms, and satisfying the relevant bond angle and dihedral angle in each case (atoms (5) and (7) in  **Figure Ac in S2 File**). For each $N,C_{\alpha}, C$ triplet, a carbonyl $O$ atom (4) was positioned so that it lay in the plane of the three atoms and formed a bond angle of $\theta_{C_{\alpha}\mathrm{CO}}=\left( \theta_{NC_{\alpha}C}-\pi\right)/2$. The protein backbone was extended in this way until the chain was complete.

For residues in the coiled-coils where there was no experimental data and for those found at the extreme edges of the crystal structure data (which may be unreliable due to the possibility of denaturing) we assumed an ideal coiled-coil structure and assigned bond angle values of $\theta_{\mathrm{NC}_{\alpha}C}=70.5^{\circ}$, $\theta_{C_{\alpha}\mathrm{CN}}=64^{\circ}$ and $\theta_{CNC_{\alpha}}=58^{\circ}$ and dihedral angle values of $\phi=-57^{\circ},\psi=-47^{\circ}$ and $\omega=180^{\circ}$.

The next stage was to add the sidechain atoms to the peptide backbone. A set of template residue sidechain coordinates were obtained from TINKER.^3^ The first sidechain atom, $C_{\beta}$, was was added to the peptide using the bond length ${|C}_{\alpha}-C_{\beta}| =$ 1.51 Å, the bond angle $\theta_{NC_{\alpha}C_{\beta}}=\theta_{NC_{\alpha}C}$ and the dihedral angle $= - 2 / 3$ (see atom (6) of **Figure Ac in S2 File**). The additional sidechain atoms were then positioned via a translational and rotational transformation of the template structure, such that the C position and the unit vectors $\mathbf{N}\mathbf{C}_{\boldsymbol{\alpha}}$ and $\mathbf{C}_{\boldsymbol{\alpha}}\mathbf{C}$ mapped onto the keratin peptide under development.

Once the sidechains had been added, the individual K1 and K10 peptide chains needed to be repositioned relative to one another to obtain the complete subdomain. To achieve this, each K1 and K10 chain was translated and rotated in space so that the backbone N atom of the N- and C-term residues matched the corresponding atom positions of the vimentin reference structure. The peptide chain was then rotated about its primary axis so that the backbone N atom of the 10th residue along the chain matched the corresponding atom in the reference structure. This ensured that each peptide chain followed the same path as the vimentin reference chain and the residues of the α-helices of the coiled-coil matched the same heptad position as vimentin, such that the sidechains pointed out in the correct directions.

At the end of this procedure, the outcome was two sets of coordinates (the K1 and K10 chains) for each of the four coiled-coil fragments, the primary sequence of which corresponded to the 1A, 1B, 2A-L2-2B (to residues K1:414/416 and K10:381/383) and 2B (from residues K1:415/417 and K10:382/384) coiled-coil subdomains of the K1/K10 dimer and the secondary structure of which corresponded to that from the vimentin dimer crystal structure.

When bringing together the two peptide chains into a coiled-coil we take no account of where the sidechains are with respect to one another, hence this resulted in some steric clashes between the sidechains that could not be overcome by simple energy minimization. Thus, three pairs of inter-chain aromatic residues (residues K1:194, 266 and 482) were manually repositioned so that the sidechains did not clash with the adjacent aromatic residue found in K10.

To generate the full rod domain structure these coiled-coil subdomains then needed to be spatially translated, reoriented and then linked together using the residue sequences of the linker regions (see  **Figure Ad in S2 File**). As K1 and K10 contain different numbers of residues in the linker domains, the challenge lay in defining a structure for the linker chains that would start and end in the correct position relative to the neighboring coiled-coil subdomains. For this reason for each chain we chose a planar loop structure consisting of a series of extended segments ( $= -120, = 115$° and $= 180^{\circ}$), two $90^{\circ}$ right-angle turns (across two residues, to start and end the loop) and a $180^{\circ}$ turn (across either 3 or 5 residues) (see **Figure Ad in S2 File**). The number of residues in each loop was varied (the L1 subdomain contained either 12 or 14 residues and the L12 linker contained 17 or 16 residues) to ensure that each end of the linker was adjacent to the appropriate coiled-coil subdomains.

# Equilibration of the dimers

To gain meaningful statistics on the dimer we needed to ensure that we only took measurements from the equilibrated structures of our models. We measured the root mean square displacement (RMSD) of the backbone atoms of the dimer, by aligning the dimer structure at time $t$ against that at time $t = 0$ and then measuring the RMSD. The model was assumed to have equilibrated for times of the trajectory data where the RMSD had plateaued.

Typically the truncated dimer models showed a plateau in the RMSD by 50 ns. In a few cases the RMSD took longer to plateau. To investigate the cause, we measured the RMSDs of the individual head, 1A and 1B domains. We found that the RMSD of each domain had plateaued within 50 ns, see **Figure Ba in S2 File**, which suggested that the structure of these domains was no longer changing. Thus any remaining changes in the RMSD of the complete structure after this time were due to reorientation between the subdomains.

To investigate this reorientation between subdomains (in particular between the head domain and 1A subdomain) we ran one example of the truncated dimer (the case that had an initially extended head domain, *i.e.* in the E category, see Methods Section and **Fig. 1C** in main text) for an extended time period of 280 ns, see **Figure Bb in S2 File**. This shows several plateaus in the RMSD, which correspond to different orientations between the head and rod subdomains (snapshots of the dimer for these are shown in **Figure Bb in S2 File**). Reorientation between the head domain and the rod domain was achieved without any interaction between the N-term residues of the head domain and the rod domain, where these residues remained retracted away from the rod domain. Hence this reorientation must have been driven by the interaction between the parts of the head facing the rod domain (*i.e.* either from those head residues sequentially next to the rod domain, as found in the H1 subdomain, or from the residues in the main globule part of the head that face the rod domain, as found in the V1 subdomain). This example demonstrated that our models could sample a variety of the possible head-rod orientations.

For the full dimer models the RMSD took much longer to plateau than was found for the truncated dimer models. **Figure Ca in S2 File** shows an example, along with insets of the dimer structure at particular times. Again we measured the RMSD of each individual dimer subdomain. The tail, head and upper rod domains (1A and 1B) equilibrated in a similar time as found for the truncated dimer. Thus, we found that the primary reason for the large change in the RMSD was from the restructuring that occurred within the 2A-L2-2B subdomains of the lower rod domain, see **Figure Cb in S2 File**. Here, from an initially identical structure the final RMSD had 20 Å range in values. On these models the most apparent structural change seen during this longer equilibration period was the development of a kink in the α-helices of the 2B chains (which is discussed further in the Rod domain structure Section of the Results in the main text).

# Ensemble properties of the dimer models

The fiber-like appearance of keratin *in situ* could imply that the dimer is rod-like. Nevertheless, the subdomain structure of the keratin rod domain, with its highly structured coiled-coils divided by less structured linkers, suggests the dimer is actually more chain-like with there being some flexibility between the coiled-coil links. Thus, to get a measure of how rod-like the rod domain of the dimer is, we calculated the planar ‘hinge’ angle ( ) made between the vectors of the two major-axes of adjacent coiled-coil subdomains (see Methods Section of the main text for details). A schematic of these angles is given in **Figure Da in S2 File**. An average hinge angle of much less than $180^{\circ}$ shows that the neighboring coiled-coil subdomains were not in parallel alignment but instead had the in-between linker subdomain acting like a hinge. When the hinge angle is less than $90^{\circ}$the coiled-coil subdomains were angled towards one another.

**Figure Db in S2 File** shows that the distribution of the hinge angle made between the 1A and 1B rod domains is doubled peaked. This suggests that there are two preferred orientations: (i) that the 1A and 1B rod domains were bent obtusely at $45^{\circ}$from parallel; or (ii) they were bent at right angles to one another. By contrast, the planar hinge angle between the 1B and 2A subdomains and the 2A and 2B subdomains are single-peaked with a single equilibrium position, at $137^{\circ}$(*i.e.* these domains were obtusely bent at $45^{\circ}$ from parallel) and $170^{\circ}$ (close to parallel), respectively. These results indicated that overall the dimer rod domain assumed a bracket-shaped, chain-like profile (see examples in **Fig. 4A** of main text), with the L1 and L12 subdomains acting as hinges, rather than behaving as rod-like.

We next tested how bent the central axis of each of the coiled-coil domains was (see **Figure Da in S2 File** for a schematic). The planar ‘bend’ angle of the coiled-coil domain () was defined as the angle between the two local vectors that point in the direction of the coiled-coil at each end of the relevant subdomain. The smaller the angle of the more bent the domain was, where an untwisted rod-like domain would have $= 180^{\circ}$. **Figure Dc in S2 File** shows the distribution of the bend angle for each domain. Subdomain 2A was not included as it was always found to give $= 180^{\circ}$(*i.e.* it behaved like a straight rod). The mean bend angles were calculated as $162^{\circ}$ (full dimer only)/$157^{\circ}$(all models) for the 1A subdomain, $137^{\circ}$ for the 1B subdomain and $141^{\circ}$ for the 2B subdomain. Hence, both the 1B and 2B subdomains were curved at $40^{\circ}$ (with a standard deviation of 20°) from straight while the 1A subdomain was much straighter with only a $20^{\circ}$ deviation (and a standard deviation of $10^{\circ}$) from linear. Thus overall the dimer profile may be slightly curved.

The head domain contains more residues (a total of 326 residues) than the tail domain (270 residues). To measure the globule size of head and tail we calculated their radius of gyration $R_{\text{gyr}}$ (see Method Section of main text for definitions). The histograms of $R_{\text{gyr}}$, see **Figure Dd in S2 File**, show that the head domain had a $R_{\text{gyr}}$ between 20–35 Å with mean of 25.7 Å (or 0.078 Å per head residue) and standard deviation of 4.3 Å. When only the residues contained within the globular V1 head subdomain are considered, the radius of gyration decreases to 18.7 Å with a standard deviation of 2.6 Å indicating a more compact structure. The histograms of $R_{\text{gyr}}$ for the tail domain had two populations (centered at ~17 Å and ~22 Å) and a mean of 21.5 Å (or 0.079 Å per tail residue) and standard deviation of 3.6 Å.

Next, the end-to-end distance was measured between the $C_{\alpha}$ atoms of the first and last residue of each chain in the head and tail (definitions given in Method Section of main text). **Figure De in S2 File** shows the distribution of end-to-end distances for the head and tail. Several peaks are seen in the histograms which broadly correspond to whether the N-term or C-term of the head and tail were attached to the rod (larger values) or the globule part of these domains (lower values). We found that the end-to-end distances of the head domain had a mean of 50.6 Å (or 0.28 Å per residue) and a standard deviation of 21.3 Å for the K1 chain, and a mean of 42.9 Å (or 0.29 Å per residue) and standard deviation of 12.2 Å for the K10 chain. The tail domain featured end-to-end distances with a mean of 39.3 Å (0.25 Å per residue) and standard deviation of 23.7 Å for K1, and mean of 45.0 Å (or 0.36 Å per residue) and standard deviation of 12.0 Å for the K10.

# Dividing the head and tail domains into a tri-domain structure using keratin informatics data

In the main text we propose a tri-domain structure for the head and tail domain of the dimer based on the structural features of our models. To determine the residue boundaries of the subdomains we identified the highly conserved parts of the primary sequence [4-5]. **Figure E in S2 File** gives the sequence alignment for several epithelial type II keratin proteins. The highly conserved parts of the sequence were residues K1:61-82 (which we denoted as C1), 144-153 (C2), 154-168 (C3) and 169-180 (C4). Similar but less conserved sequence stretches were found for the type I keratins, see **Figure Ea in S2 File**, at residues K10:31-40 (D1) and 138-146 (D2). In the tail domain only the type II keratin shows conservation, found between residues K1:494-513 (C5), (see **Figure Eb in S2 File**).

By overlaying the residue boundaries of these sequence motifs onto our contact plots, shown in **Figs. 5A** and **6A** of the main article as dotted boundary lines, we were able to divide the head domain into several distinct regions. By doing this we found that the residues K1:144-180, which had been considered to be of one subdomain, were better described divided in two subdomains, due to the differences in the number and extent of contacts present, where a PRO residue resides at the boundary. Here residues K1:144-153 appeared partly buried in the V1 head domain structure. This can be seen in our schematics of the head domain, see **Fig. 2A** in main text, where these residues are highlighted in green.

# Sidechain Contacts

We measured the number and types of residue sidechain contacts present in the dimer. The side chain contacts were classified depending on the type of residue present (as given by the residue pairing matrix in **Figure Fc in S2 File**). To avoid double counting only one type of interaction was measured for each sidechain pairing which potentially involved two or more types of interactions. Contacts were said to be present if the distance between a pair of sidechain reference atoms was less than contact type dependent cutoff distance (these values are given in tables in **Figure Fc in S2 File**). For the pairings between the four polar residues SER, THR, GLN and ASN both potential sites for hydrogen bonding were checked. The total number of contacts were calculated by summing over the K1-K1, K1-K10 and K10-K10 contacts.

**Figure** **F in S2 File** presents the average number of sidechain contact pairings within key subdomains and between subdomains. The overall characters of the head, tail and rod domains were found to be different with aromatic ring contacts being much more prevalent in the head and tail domain than in the rod domain and hydrophobic contacts being more important in the rod domain. Overall we measured 604 (1.0 per residue), 174 (0.5 per residue) and 107 (0.4 per residue) potential sidechain contacts in the rod, head and tail domains, respectively. The presence of fewer intra-tail sidechain contacts suggests that sidechain-sidechain interactions play less of a role in the tail compared to intra-head interactions and may explain why the tail appears more mobile in the simulations.

Electrostatic interactions made only a small contribution to the head and tail, numbering an average total of 5 (head) and 4 (tail), but a more significant contribution of 47 pairs in the rod domain. Acid-base pairing in the head domain could only have come from within the H1 subdomain or between the H1 and V1 subdomains, due to the sequence distribution of acidic and basic residues in the head domain. Larger contributions from electrostatic interactions are seen between domains such as across the L1 linker (averaging 5 contacts) and between head and rod domains (averaging 9 contacts), but not across the L12 (2 contacts) or tail-rod (2 contacts), and were important for stabilizing the hinge angle between these domains.

Our results also showed that it was very rare to find sulfur atoms close to one another in the dimer (with an average of less than one occurrence).

The high number of aromatic interactions found in the head domain (totaling 21 pairs from the 38 aromatic residues found in the head domain) indicates that on average each aromatic ring was interacting with at least one other. This is consistent with the occurrence of a network of interconnected glycine loops. In the tail domain fewer aromatic pairings were found (with 7 pairs found from the 17 aromatic residues) and from this we infer that these glycine loops were less interconnected. A large number of polar-aromatic pairings was also found in these domains, with around 77 (head) and 61 (tail) pairings found and was much larger than the polar (acid/base)-polar interactions which totaled 25 (head) and 23 (tail).

# Hydrogen and Hydrogen-π Interactions

To further characterize the polar-polar and polar-aromatic pairings we determined the number of hydrogen and hydrogen-π bonds present, which could involve both backbone and sidechain atoms. A hydrogen bond was said to be present if the distance between the polar hydrogen (H) and acceptor heavy atom (A) was less than 3.5 Å and the angle X-H--A was less than $30^{\circ}$, where X is the donor heavy atom attached to H. Hydrogen bonds that were part of salt bridges (electrostatic) were not included. A hydrogen-π bond was said to be present if the distance between the donor heavy atom, attached to the polar hydrogen atom, and the center of the aromatic ring was less than 4.3 Å and the angle between the vector normal to the plane of the aromatic ring and the vector from the center of the ring to the donor heavy atom was less than $25^{\circ}$ (cutoff distances were obtained from Steiner & Koellner [6]). Details on the hydrogen donor used are given in **Figure G in S2 File.**

**Figure G in S2 File** shows the distribution of the number of hydrogen and hydrogen-π bonds found in each frame. We divided the interactions into groups depending on the dimer domains and monomer chains involved and whether the hydrogen bonding pair of atoms was from the peptide backbone, residue sidechains or between a sidechain and the backbone. Despite the globular appearance of the head and tail domains, the majority of the hydrogen bonds in these domains were due to backbone-backbone or backbone-sidechain interactions. This is due to the large amounts of glycine and serine, which make up most of these domains. The head domain has many more inter-chain hydrogen bonds than the tail and these may help keep the head as one globular structure. Similar results were found for hydrogen-π bonding in the head and tail domain, which was predominantly between the backbone and the aromatic ring of the same peptide chain.

We calculated the average number of hydrogen bonds present in each domain, see **Figure G in S2 File**, and found that there were on average 93 head (85% involve the backbone), 32 tail (92% involve the backbone) and 271 rod (80% involve the backbone) hydrogen bonds and 6 head (60% involve the backbone), 4 tail (22% involve the backbone) and 3 rod (28% involve the backbone) hydrogen-π bonds. Both of these counts have smaller contributions from sidechains than might have been expected from the number of sidechain contacts measured and suggested that specific sidechain-sidechain alignment that results in hydrogen bonding occurs infrequently.

Together these data suggest that the aromatic rings in the head and tail may be key to holding the globules together though the formation of glycine loops *via* interactions between aromatic rings, between polar hydrogen atoms attached to the backbone and the aromatic ring and through hydrogen bonds between the backbones of the glycine loops.

# Sulfide Residues

The keratin dimer contains 7 cysteine residues (4 in the Head domain, 2 in the 2B subdomains and 1 in the tail domain). Out of these, only the cysteines in the head domain (K10: 25, 66 and K1: 49, 142) may be suitable for potentially forming disulfide bridges within the K1/K10 keratin. However, the three dimensional structure of this domain is insufficiently known to assign disulfide bonds to our models.

From our simulation results, the correlation in cross-chain residue contacts found for the head domain (see **Fig. 5** of the main text) suggests that a disulfide bond between E1 subdomain’s K10:25 and K1:49 may be possible and this could further aid in holding the K1 and K10 chains together in one globular structure.

# Supplementary references

1. Nicolet S, Herrmann, H Aebi, U, Strelkov, SV. Atomic structure of vimentin coil 2. Journal of Structural Biology 2010; 170: 369-376.

2. Strelkov SV et al. Divide-and-conquer crystallographic approach towards an atomic structure of intermediate filaments. Journal of Molecular Biology 2001; 306: 773-781.

3. Ponder JW et al. TINKER - Software Tools for Molecular Design. 6.2 edn (Washington University School of Medicine, 2013).

4. Kimonis V,  DiGiovanna JJ, Yang J-M, Doyle SZ, Bale SJ, Compton JG. A Mutation in the V1 End Domain of Keratin 1 in Non-Epidermolytic Palmar-Plantar Keratoderma. J Investig Dermatol 1994; 103: 764-769.

5. Steinert PM, Parry DA. The conserved H1 domain of the type II keratin 1 chain plays an essential role in the alignment of nearest neighbor molecules in mouse and human keratin 1/keratin 10 intermediate filaments at the two- to four-molecule level of structure. Journal of Biological Chemistry 1993; 268: 2878-87.

6. Steiner T, Koellner G. Hydrogen bonds with pi-acceptors in proteins: Frequencies and role in stabilizing local 3D structures. Journal of Molecular Biology 2001; 305: 535-557.
