# Supplementary material for: Complete Structure of an Epithelial Keratin Dimer: Implications for Intermediate Filament Assembly
Source: PLoS One. 2015 Jul 16;10(7):e0132706. doi: 10.1371/journal.pone.0132706 (PMC4504709; doi:10.1371/journal.pone.0132706)
Supplement: S2 File — Fig A, Keratin model building. Fig B, Evolution in structure of the truncated dimer models. Fig C, Evolution in structure of the full dimer models. Fig D, Statistical geometry of the dimer. Fig E, Definition of the subdomains of the head and tail. Fig F, Contacts between amino acid side chains. Fig G, Details on Hydrogen-bond interactions. (DOCX) [file pone.0132706.s002.docx]

S2 File: Supplementary Figures for the Manuscript Entitled:

Complete Structure of an Epithelial Keratin Dimer: Implications for Intermediate Filament Assembly

David J. Bray^1,2^, Tiffany R. Walsh^3*^, Massimo G. Noro^4^, Rebecca Notman^1,2*^

^1^Department of Chemistry University of Warwick, Coventry, UK

^2^Centre for Scientific Computing, University of Warwick, Coventry, UK

^3^Institute for Frontier Materials, Deakin University, Geelong, VIC, Australia

^4^Unilever R&D Port Sunlight, Wirral, UK

*Corresponding author

Email: [r.notman@warwick.ac.uk](mailto:r.notman@warwick.ac.uk) (RN); tiffany.walsh@deakin.edu.au (TRW)

**Figure A. Keratin model building.**

(a) Higher-order organization of keratin highlighting the four key tetramer arrangements (A_11_, A_22_, A_12_, A_CN_).

(b) Differences between the monomer (based on previous sequence analysis) and dimer (based on our structure analysis) subdomain structure of keratin.

(c) Key stages of model construction of the rod domain of K1/K10 based on the structure of vimentin. Atoms are labelled 1 to 8 for identification in Section B in S1 File. The three principal backbone dihedral angles, *φ*, *ψ* and *ω*, and sidechain dihedral angle *χ* are also shown.

(d) Initial loop structure of peptide chains of the L12 linker. The number of residues in each segment are given in italics.

(e) Initial setup of the U-shaped chains of the head and tail domain. Parameter *n* indicates the shift in residue value of the beginning of the hairpin turn in these chains. The letters indicate the geometry of the two chains: configurations **A** and **B** have the head and tail domains folded back towards the rod domain in the same direction; **C** and **D** have the K1 and K10 chains of the head and tail domain folded onto opposite faces of the rod; and **E** has no hairpin turn and the chains are extended away from the rod domain.

(f) Example of an initial configuration of the dimer showing the folded head and tail domain, the coiled-coil structures based on vimentin of the 1A, 1B and 2A-L2-2B subdomains and the loop structures of the L1 and L12 linker subdomains.

**Figure B. Evolution in structure of the truncated dimer models.**

(a) Root mean square deviation (RMSD) of the head domain and 1A and 1B subdomains.

(b) The RMSD of the truncated dimer with an initially unfolded head domain and corresponding snapshots of the dimer structure.

**Figure C. Evolution in structure of the full dimer models.**

(**a**) Root mean square deviation (RMSD) of the full dimer with an initially folded head group and corresponding snapshots of the dimer structure.

(**b**) RMSD of the 1B, 2A and 2B subdomains and tail domain.

**Figure D. Statistical geometry of the dimer.**

(a) Schematics showing an example of the measured angles, radius of gyration *R*_gyr,_ measured from centre of mass x_com;_ and the end-to-end distance of each chain, *d*.

(b) Distribution of the hinge angle, between the major coiled-coil rod domains.

(c) Distribution of the bend angle in the major coiled-coil rod domains.

(d) Radius of gyration for the head and the tail domains.

(e) End-to-end distances, *d*, of the head and tail domains for each chain

**Figure E. Definition of the dimer’s tri-subdomain head and tail.**

1. Break down of primary sequence in the head domain into the E1, V1, H1 subdomains.
2. Break down of primary sequence in the tail domain into the E2, V2, H2 subdomains.

Highly conserved segments of the sequences (denoted *C1-5, D1-2*) are used to help define domain boundaries. At the bottom of each table the subdomain structure of the keratin monomer is given.

**Figure F Contacts between amino acid side chains.**

(a) Type and number of inter-domain sidechain contacts.

(b) Type and number of intra-domain sidechain contacts.

(c) Details on parameters used when measuring contacts.

**Figure G. Details on Hydrogen-bond interactions.**

(a) Number of backbone-backbone hydrogen bonds.

(b) Number of sidechain-sidechain hydrogen bonds.

(c) Number of backbone-sidechain hydrogen bonds.

(d) Number of hydrogen-π bonds.
